# Engineering G protein‐coupled receptors for stabilization

**DOI:** 10.1002/pro.5000

**Published:** 2024-05-15

**Authors:** João Paulo L. Velloso, Alex G. C. de Sá, Douglas E. V. Pires, David B. Ascher

**Affiliations:** ^1^ School of Chemistry and Molecular Biosciences, The Australian Centre for Ecogenomics The University of Queensland Brisbane Queensland Australia; ^2^ Computational Biology and Clinical Informatics Baker Heart and Diabetes Institute Melbourne Victoria Australia; ^3^ Baker Department of Cardiometabolic Health The University of Melbourne Parkville Victoria Australia; ^4^ School of Computing and Information Systems The University of Melbourne Parkville Victoria Australia

**Keywords:** G protein‐coupled receptors, machine learning (ML), protein engineering, stability prediction, structural characterization

## Abstract

G protein‐coupled receptors (GPCRs) are one of the most important families of targets for drug discovery. One of the limiting steps in the study of GPCRs has been their stability, with significant and time‐consuming protein engineering often used to stabilize GPCRs for structural characterization and drug screening. Unfortunately, computational methods developed using globular soluble proteins have translated poorly to the rational engineering of GPCRs. To fill this gap, we propose GPCR‐tm, a novel and personalized structurally driven web‐based machine learning tool to study the impacts of mutations on GPCR stability. We show that GPCR‐tm performs as well as or better than alternative methods, and that it can accurately rank the stability changes of a wide range of mutations occurring in various types of class A GPCRs. GPCR‐tm achieved Pearson's correlation coefficients of 0.74 and 0.46 on 10‐fold cross‐validation and blind test sets, respectively. We observed that the (structural) graph‐based signatures were the most important set of features for predicting destabilizing mutations, which points out that these signatures properly describe the changes in the environment where the mutations occur. More specifically, GPCR‐tm was able to accurately rank mutations based on their effect on protein stability, guiding their rational stabilization. GPCR‐tm is available through a user‐friendly web server at https://biosig.lab.uq.edu.au/gpcr_tm/.

## 1 INTRODUCTION

G protein‐coupled receptors (GPCRs) are members of one of the most medicine‐relevant human protein families (Hauser et al., 2017). They possess the ability to selectively bind to a diverse range of ligands, including light‐sensitive compounds, pheromones, hormones, ions, and neurotransmitters. These receptors then transmit these signals from the external environment of the cell to its intracellular side. In 2017, it was estimated that around 34% of all drugs approved by the United States Food and Drug Administration targeted GPCRs. This corresponds to approximately 700 approved drugs (Hauser et al., 2017). Nevertheless, the majority of GPCRs were unexplored, and there are approximately 227 non‐olfactory GPCRs that are yet to be analyzed by drug discovery processes (Hauser et al., 2017).

Despite all the recent discoveries around GPCRs, we still have many structures to be discovered. According to the GPCRdb (Pandy‐Szekeres et al., 2023), 187 unique structures were elucidated, out of 800–1000 GPCRs (Alhosaini et al., 2021; Congreve et al., 2020). In this context, the emergence of cryo‐electron microscopy (cryo‐EM) has revolutionized GPCR structural biology. cryo‐EM enables the visualization of GPCR structures in their native states, circumventing the need for crystallization and providing insights into diverse ligand‐bound conformations (Cheng, 2015; Frank, 2002).

Complementary to cryo‐EM, x‐ray crystallography can provide higher‐resolution structural information, which can be of great value for structure‐based drug design. Nevertheless, obtaining diffraction‐quality crystals for high‐resolution structure determination of GPCR is not a trivial task. This struggle is linked to the diversity of ligands and unique features (highly flexible and dynamic). To overcome this barrier, engineering is usually required to minimize conformational heterogeneity and maximize crystal contacts and stability (Kobilka & Deupi, 2007). For this purpose, several methodologies have been developed, which include recombinant overexpression, purification strategies (Errey & Fiez‐Vandal, 2020), crystallization platforms (Parker & Newstead, 2012), and detergent studies (Lee et al., 2020). However, these studies are primarily experimental (i.e., human‐dependent), with complex and time‐consuming methodologies, resulting in low scalability while dealing with new GPCRs.

In addition, it is worth noting that it is well known that GPCRs do not work in a simple turn‐on or off state. Instead, it is understood that GPCRs work in a spectrum of states, from which either completely active or completely inactive is almost always not the case (Park et al., 2008). It is also important to mention that the interactions between GPCR ligands and receptors can unfold in a variety of relationships. For example, they can unfold under positive and negative allosterism (May et al., 2004), inverse agonism (May et al., 2004), ligand‐biased signaling (Wootten et al., 2018), and receptor oligomerization (Milligan et al., 2019). The discovery of new GPCR structures in different states is, therefore, of paramount importance. The differences between the states can shed light on our understanding of cell signaling mechanisms and support structure‐based drug design.

A strategy deployed to engineer new stable GPCR structures is the use of point mutations. Several studies involving mutations in GPCR have already been developed. For instance, more stable neurotensin receptor mutants were obtained using a systematic mutational approach coupled with activity assays (Shibata et al., 2013). A second example of it involved the application of mutagenesis in the search for more stable adenosine A2a receptor mutants (Lebon et al., 2011). Following a different idea, mutagenesis was used to obtain detergent‐solubilized thermostable mutants of β1‐adrenergic receptors (Magnani et al., 2008; Shibata et al., 2009). We also had the application of mutagenesis to generate thermostabilized free fatty acid receptor 1 (Hirozane et al., 2014). Additionally, to aid x‐ray crystallography, thermostabilization can support the study of dynamic functional aspects, such as ligand binding kinetics and receptor activation under physiological conditions. Finally, thermostabilization facilitates high‐throughput screening assays for drug discovery efforts, complementing cryo‐EM studies in identifying novel ligands and allosteric modulators (Lee et al., 2020).

However, all these studies involving mutagenesis were complex, expensive, and time‐consuming because the number of possible sequences generated by mutagenesis (sequence space) was too high, considering the number of residues in the GPCRs. Furthermore, it is important to emphasize that these strategies have been successfully used for only a few GPCRs (Vaidehi et al., 2016).

Considerable computational‐driven efforts have been taken in crafting stability predictors for protein mutations, including DUET (Pires et al., 2014), mCSM (Pires et al., 2014), SDM (Pandurangan et al., 2017; Worth et al., 2011), DDGun (Montanucci et al., 2022), MAESTROweb (Laimer et al., 2016), Dynamut (Rodrigues et al., 2018), Dynamut 2 (Baek et al., 2021), and DDMut (Zhou et al., 2023). Although these predictors have been grounded in globular proteins, their effectiveness has been somewhat restricted in handling membrane proteins. mCSM‐membrane (Pires et al., 2020) emerged as a dedicated machine learning (ML) predictor designed exclusively for membrane proteins. However, given that GPCRs constitute a highly unique subgroup, even among membrane proteins, it is crucial to be cautious when using predictors that have not been specifically built for GPCR data.

Based on the lack of stability predictors for GPCR engineering, we developed GPCR‐tm to speed up and reduce the costs of engineering‐based mutagenesis studies within the GPCR scope. GPCR‐tm relies on currently available mutation GPCR data, leading to a robust and accurate ΔTm predictor capable of ranking and stabilizing mutations tailored for GPCRs. GPCR‐tm integrates graph‐based signatures with a range of auxiliary features, providing a straightforward, explainable, and complete analysis of the impact of mutations on a protein's dynamics and stability resulting from stability changes caused by point mutations. Our method is available as a tool through an easy‐to‐use and reliable web interface at https://biosig.lab.uq.edu.au/gpcr_tm/.

## 2 RESULTS

### 2.1 Data set analysis

We compiled experimental ΔTm data from various databases, including FireProtDB (Stourac et al., 2021), Thermomutdb (Xavier et al., 2021), and MPTherm‐pred (Kulandaisamy et al., 2021). All datasets were combined, and just one mutation was selected from repeated ones (same position, same UniProt [Coudert et al., 2022] identification, mutated to the same residue). In total, we ended up with 97 non‐redundant mutations. The distributions of the stability value in the 97 single‐point mutations show that 60 mutations cause an increase in stability higher than 0. Figure S1 depicts the distribution of ΔTm values for the collected GPCRs.

Our training dataset comprises data from 11 distinct receptors (Uniprot IDs: O14842, P08172, P21554, P24530, P25024, P28335, P29274, P35408, P48039, P49286, and P51677), with 6 receptors included in both training and test sets (Uniprot IDs: O14842, P25024, P28335, P29274, P48039, and P49286). The selection of mutations for each set was conducted entirely at random, ensuring an unbiased approach to dataset construction. This meticulous approach underscores the integrity and reliability of our data, facilitating robust analysis and interpretation of the results. In addition, although training and test sets share receptors, it is important to reiterate that they do not have repeated mutations (i.e., non‐redundant sets in terms of mutations).

We also analyzed the distribution of different amino acid (AA) types on wild‐type and mutated residues. Most of the single‐point mutations determined through experiments involve hydrophobic residues, namely alanine, phenylalanine, leucine, and valine (53, 6, 7, and 3, respectively), followed by charged residues, namely arginine, and lysine (both 5 and 4, respectively). Most of the residues involved are mutated to Ala (Ryu et al., 2023), as a reflection of alanine scanning efforts (Munk et al., 2019).

Moreover, we observed mutations on all helices of the receptor by investigating the topology involved in those mutations. Helix 3 has the highest number of mutations, 28. Next, we have 18 mutations on Helix 2, 10 on Helix 5, 11 on Helix 6, and 10 on Helix 7. Helices 4 and 1 have the least number of mutations, 9 and 5, respectively. This trend is related to the fact that Helix 3 is central to the receptor, showing more interactions with other helices, whereas Helices 4 and 1 are more outermost, interacting much less with the other helices (Munk et al., 2019). The remaining six mutations occurred outside the helices.

### 2.2 Predictive performance on single‐point mutations

In an effort to build a robust and reliable model in GPCR‐tm for predicting the effects of single‐point mutations on GPCR structure stability, we used structures in both states (inactive and active). The selection of the best state and ML algorithm was based on a 10‐fold cross‐validation approach using Pearson's correlation coefficient as the performance metric. We concluded that the best scenario was using structures on active state and Random Forest (with 300 predictive decision trees) as the ML algorithm.

#### 2.2.1 ΔTm GPCR‐tm's performance

We generated two sets of features that are diverse and complementary (refer to Table 3). This detailed characterization served as the foundation for training, validating, and evaluating predictive supervised models aimed at forecasting stabilization in GPCR proteins induced by these mutations. Utilizing all features during cross‐validation yielded a Pearson's coefficient of 0.19 and a mean squared error (MSE) of 19.92. These results suggest that additional refinement and feature selection are required to enhance the model's predictive capabilities.

To enhance the model's performance, we implemented a bottom‐up greedy feature selection method (see Figure S2). Through this approach, we evaluated our feature set and the model's performance notably improved during cross‐validation. Specifically, the Pearson's coefficient increased to 0.74, and the MSE was 29.00, achieved with the utilization of 14 selected features (see Figure S3, 10‐fold cross‐validation scatter plot). When evaluating the ranking predictive performance, the Kendall's tau metric was 0.51, and the Spearman's rank‐order correlation coefficient was 0.67.

**FIGURE 1:**
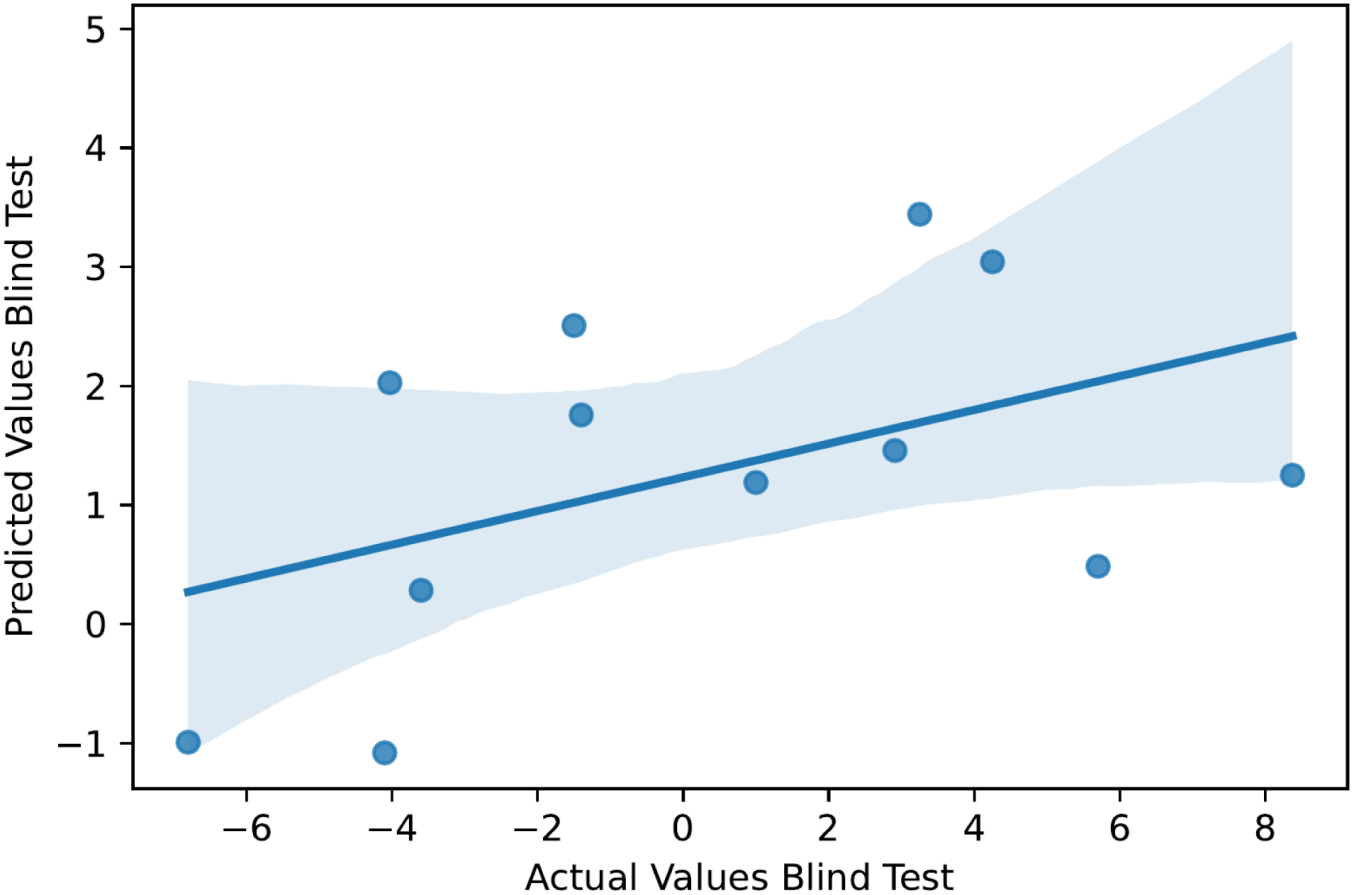
Regression analysis on G protein‐coupled receptors (GPCR)‐tm. By using the predictions on the selected (blind) test set, we externally analyzed the performance of the GPCR‐tm model. The plot demonstrates the correlation between experimental and predicted values.

Subsequently, we established a correlation between predicted and actual ΔTm values. We subjected our proposed model to a blind test, as detailed in Section 4. During this evaluation, our model yielded a Pearson coefficient of 0.46 (refer to Figure 1) and an MSE of 16.85. When evaluating the ranking performance in the blind test, the Kendall's tau metric was 0.27, and the Spearman's rank‐order correlation coefficient was 0.41. Notably, this performance stands out as the highest in the benchmark for stability upon mutations, showcasing its robust and reliable capabilities. The outcomes of the blind test further affirm the model's effectiveness in handling new data scenarios. We have also included scatter plots in Figures S4 and S5 to demonstrate the correlation between experimentally measured ΔTm values and the rank ordering of mutations predicted by our model. These scatter plots depict the relationship observed during both 10‐fold cross‐validation and the blind test. This additional analysis provides further insight into the consistency and reliability of our predictor in estimating the impact of mutations on ΔTm values across different validation scenarios.

Subsequently, we assessed our model via a second blind test set, in which mutations are stabilizing according to GPCRdb (Pandy‐Szekeres et al., 2023). The model predicted 38 mutations (76%) properly, increasing the stability of the receptor, which provides additional confidence in the generalizability and robustness of the model in GPCR‐tm.

Nevertheless, we are aware that GPCR‐tm was modeled using a small structural GPCR dataset, which constrains its ability to accurately represent the underlying GPCR mutations, thereby increasing the risk of overfitting in terms of predicting the effect of new GPCR mutations in terms of thermostability. In the context of our study, the scarcity of available GPCR data exacerbates this challenge. Despite our efforts to increase the GPCR mutation sample size, the limited dataset has led to an observed decrease in predictive performances between cross‐validation and blind testing. However, it is noteworthy that our model still demonstrates considerable reliability, as evidenced by its performance on both blind sets applied. Additionally, in comparative benchmarking against other methods (see the following subsection), our approach outperforms alternative methods, further underscoring its efficacy in predicting GPCR thermostabilizing mutations.

#### 2.2.2 (De)stabilization model's predictive performance

Furthermore, we applied the same dataset used during blind testing of our model (i.e., considering 19 independent mutations from the training set) to benchmark against other available tools using classification by regression. We employed the performance metrics accuracy, Matthew's correlation coefficient (MCC), and weighted F1 score to compare the predictive performance of GPCR‐tm against alternative methods. Methods in Supporting Information S1 detail all these performance metrics.

GPCR‐tm significantly performed as well as or better than all alternative methods (Table 1). Nevertheless, it is worth noting that we could not directly compare GPCR‐tm with a well‐established method, MPTherm‐pred (Kulandaisamy et al., 2021). For predicting using the tool MPTherm‐pred, the user needs to select as input a Protein Data Bank (PDB) file available at https://www.rcsb.org/. MPTherm‐pred does not accept as input a PDB file to be uploaded. When selecting a structure available at https://www.rcsb.org/, the structure would contain potential structural modifications (like engineered mutations). Predictions made using these structures would not be comparable with the ones used in this study. Therefore, the comparison with this alternative method was not feasible.

**TABLE 1:** Comparative performance of GPCR‐tm (in bold) across testing data sets with alternative stability predictive methods.

Method	Thresholds for neutral	Accuracy	MCC	Weighted F1 score
GPCR‐tm	−0.35 to 0.35	**0.67**	**0.46**	**0.65**
mCSM‐membrane	−0.20 to 0.20	0.67	0.45	0.62
DUET	−1.85 to 1.85	0.33	0.16	0.23
mCSM	−1.85 to 1.85	0.33	0.16	0.23
SDM	−1.75 to 1.75	0.42	0.22	0.37
DDGun	−1.00 to 1.00	0.25	0.07	0.27
MAESTROweb	−1.85 to 1.85	0.42	0.33	0.36
Dynamut 2	−1.80 to 1.80	0.33	0.13	0.12

Abbreviation: MCC, Matthew's correlation coefficient.

Although mCSM‐membrane performed as well as GPCR‐tm on the blind test set, we believe that is because part of this data were used to build mCSM‐membrane's model, not allowing a true comparison to this alternative method. In fact, when the whole dataset with 97 proteins is set as input, the predictive performance of the mCSM‐membrane method drastically decreased, achieving an accuracy of 0.32, an MCC of −0.03, and a weighted F1 score of 0.27. These results highlight why personalizing a ML‐based tool for GPCR stabilization prediction is crucial, which is an aspect delivered by GPCR‐tm.

### 2.3 Interpretation of the selected features for predicting stabilization for GPCR mutations

**FIGURE 2:**
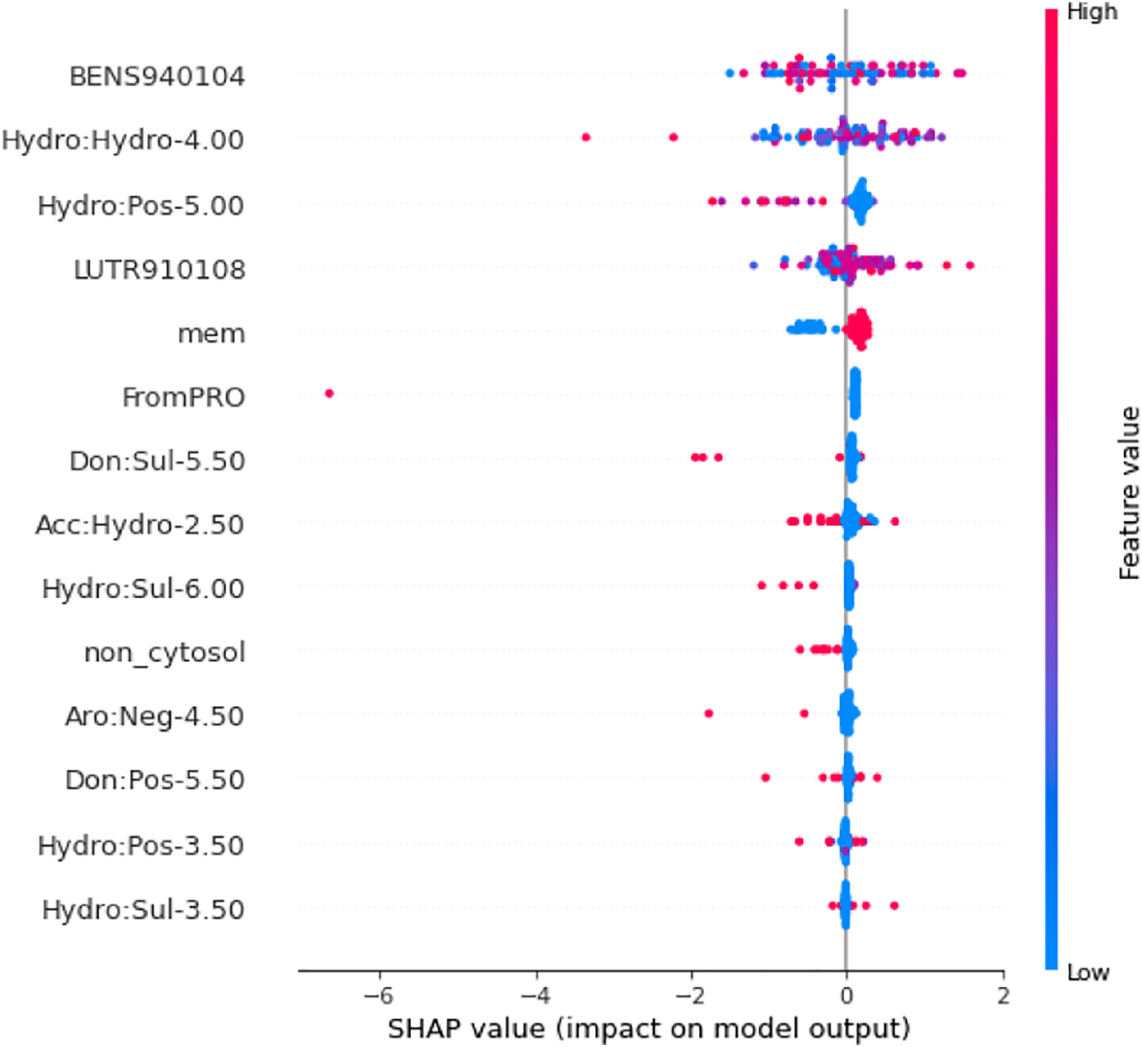
Shapley Additive Explanations (SHAP) feature importance plot. The features that compose the regression model of G protein‐coupled receptors‐tm are represented in descending order of importance on the vertical axis. The points represent the training data set. High values are represented in red color, while points with low values are presented in blue. Points to the left influenced the prediction to be destabilizing (negative values), and points to the right, stabilizing (positive values). Different input features affect the output of the respective model (regressor).

During feature selection of GPCR‐tm (regressor tool), we found that out of 14 features, 9 graph‐based signatures were important for ranking mutations (see Figure 2; Table S1). Graph‐based signatures represent the quantification of pairs of pharmacophoric regions within a defined distance threshold surrounding the mutation site. For example, the feature denoted as Hydro:Hydro‐4.00 indicates the count of pairs of hydrophobic atoms within a maximum distance of 4 Å from the mutation site. Refer to Section 4 for more information about graph‐based signatures. Six out of the nine graph‐based signatures are related to hydrophobic interactions: Hydro:Hydro‐4.00, Acc:Hydro‐2.50, Hydro:Pos‐5.00, Hydro:Sul‐3.50, Hydro:Sul‐6.00, and Hydro:Pos‐3.50 (where Hydro = hydrophobic, Acc = hydrogen bond acceptor, Pos = positive, Sul = sulfur group, and the given number represents the distance cutoff in angstroms). This selection can be related to the fact that membrane proteins, like GPCRs, are embedded in a hydrophobic environment (membrane). Therefore, the hydrophobic interactions play an important role in membrane insertion and folding (Ballesteros & Weinstein, 1995). Any changes in the hydrophobic core can cause the protein to lose stability. According to the Shapley Additive Explanations (SHAP) (Lundberg & Lee, 2017) feature importance plot in Figure 2, when the values of the features Hydro:Pos‐5.00, Acc:Hydro‐2.50, and Hydro:Sul‐6.00 are high, the predictions tend toward not stabilizing. For the feature Hydro:Hydro‐4.00, there is no visible pattern. In this case, high and low values are correlated to stabilizing and destabilizing. Another important aspect that can be observed is related to the feature mem (stands for membrane; if its value is high, the residue where the mutation occurred resides inside the membrane; if its value is low, the residue where the mutation occurred resides outside the membrane). According to the SHAP plot, mutations inside the membrane are correlated to an increase in stability, and mutations outside the membrane are correlated to a decrease in stability. We also found four features related to charged residues (Don:Pos‐5.50, Hydro:Pos‐5.00, Hydro:Pos‐3.50, and Aro:Neg‐4.50, where Don indicates a hydrogen bond donor), which reflects the importance of electrostatic interactions for protein stability (Hassani, 2012; Matthews, 1993; Pace et al., 2000) (refer to Table S1, for more information about each feature).

The features BENS940104 (i.e., based on the genetic code matrix) (Benner et al., 1994) and LUTR910108 (i.e., based on the structure‐based comparison table for alpha helix class) (Luthy et al., 1991) have also been considered important in our findings (see Table S1). Their importance is related to the fact that they help in identifying regions of proteins that are prone to mutation and those that are evolutionarily conserved, which can provide insights into functional regions and structural stability. BENS940104 feature is based on a genetic code matrix. The matrix is calculated by assuming that the genetic code is the only constraint on AA divergence. A higher value of this feature indicates that the observed AA substitution occurs more frequently than expected by chance, while a lower value indicates that the substitution occurs less frequently than expected by chance. When analyzing the SHAP feature importance plot (Figure 2), relating the value to the impact of it on the prediction is unclear. Nevertheless, higher values are apparently more related to a decrease in stability. This indicates that these destabilizing mutations have a higher frequency, according to this matrix. Conversely, the LUTR910108 feature is based on a secondary structure‐based profile made for mutations occurring in alpha helices. Lower values meaning that the mutation tends to occur less often when happening in an alpha helix. Higher values mean the opposite. According to the SHAP feature importance plot (Figure 2), the higher values of this feature tend to indicate an increase in stability.

The feature non_cytosol (i.e., a mutation not inserted into the cytosol) provides information about the location of the mutation. This is critical for the prediction because the cytosolic environment is completely different from the regions outside of the cytosol in terms of lipophilicity and substances concentrations (Kulandaisamy et al., 2019). The feature FromPro refers to mutations in which the original AA is a proline. This is biologically relevant because this is an AA that has a distinctive cyclic side chain. This special side chain gives proline an exceptional conformational rigidity compared to other AAs. Proline is often found in turns, loops, and bends within protein structures. The change of proline to another AA commonly leads to a decrease in structural rigidity (Choi & Mayo, 2006) (see Table S1 for more information).

### 2.4 GPCR‐tm's web server

GPCR‐tm is available as a user‐friendly web server. To perform a prediction, users need to provide a PDB file or a PDB code of the GPCR. They can upload a list of mutations. The point mutation should contain the single‐letter code of the wild‐type residue, the corresponding mutation residue number, and the single‐letter code of the mutant residue. The chain identifier of the wild‐type residue in the protein should also be specified in its single‐letter code. The single‐letter code and the chain identifier should be separated by a space.

GPCR‐tm predicts ΔTm, which is a metric related to how a single‐point mutation will affect protein stability. According to the ΔTm, the mutations are ranked following a descending order of ΔTm on GPCR‐tm's web server. The higher the result, the higher the probability of the mutation increasing the stability of the receptor. The results can be downloaded in a tabular comma‐separated value format (Figure S6).

## 3 CONCLUSIONS

Here, we present GPCR‐tm, a ML web‐based method, GPCR‐tm, which relies on the concepts of graph‐based signatures and auxiliary features to predict and rank the effects of single‐point missense mutations on the stability of GPCRs. This is the first approach designed exclusively for GPCRs, which incorporates a user‐friendly web server for seamless interaction.

GPCR‐tm could accurately predict the effects of a variety of mutations on many different types of GPCRs on two non‐redundant sets, which guarantees the robustness and reliability of our method. One downside related to the availability of data is that it was trained and assessed using class A GPCR information only. Because of the structural differences between other classes, there is no guarantee that the performance demonstrated through our work is transferable to other classes.

It is also crucial to acknowledge that the limited data availability posed challenges during the GPCR‐tm model's development. Nevertheless, we benchmarked our proposed method against alternative methods, showing that GPCR‐tm outperformed or performed similarly to these methods. Hence, GPCR‐tm represents a significant advance over current ranking platforms, which have been built for GPCRs.

We would also like to stress the relationships between GPCR‐tm and GPCR structural elucidation studies. As cryo‐EM caused a revolution in the GPCR structural biology field, it offered an alternative approach to traditional crystallography methods that often require protein engineering through point mutations to develop more stable proteins. Additionally, it is also important to point out that thermostabilizing mutations may be needed to just facilitate purification and structure determination but are not usually required once the structure has been determined. Despite that, thermostabilizing GPCRs using point mutations is still beneficial for several reasons. For example, it supports the study of dynamics and functions, such as ligand binding kinetics and receptor activation. Additionally, thermostabilization facilitates high‐throughput screening assays for drug discovery efforts, complementing cryo‐EM studies in identifying novel ligands and allosteric modulators. Moreover, GPCR‐tm benefits from cryo‐EM GPCR structures and any other tool that supports structure elucidation, as the model relies on a structure for ranking mutations based on their potential to enhance structural stability.

GPCR‐tm is intended to support mutagenesis studies for GPCRs, decreasing the time and expenses of those studies. The model was built and validated using AlphaFold‐Multistate models, displaying its reliability even when utilizing AlphaFold models for mutation prediction. GPCR‐tm offers a robust ΔTm predictor, ranking stabilizing mutations tailored for GPCRs. This proposed tool is freely available as a scalable, user‐friendly, and easy‐to‐use web server at https://biosig.lab.uq.edu.au/gpcr_tm/.

## 4 METHODS

**FIGURE 3:**
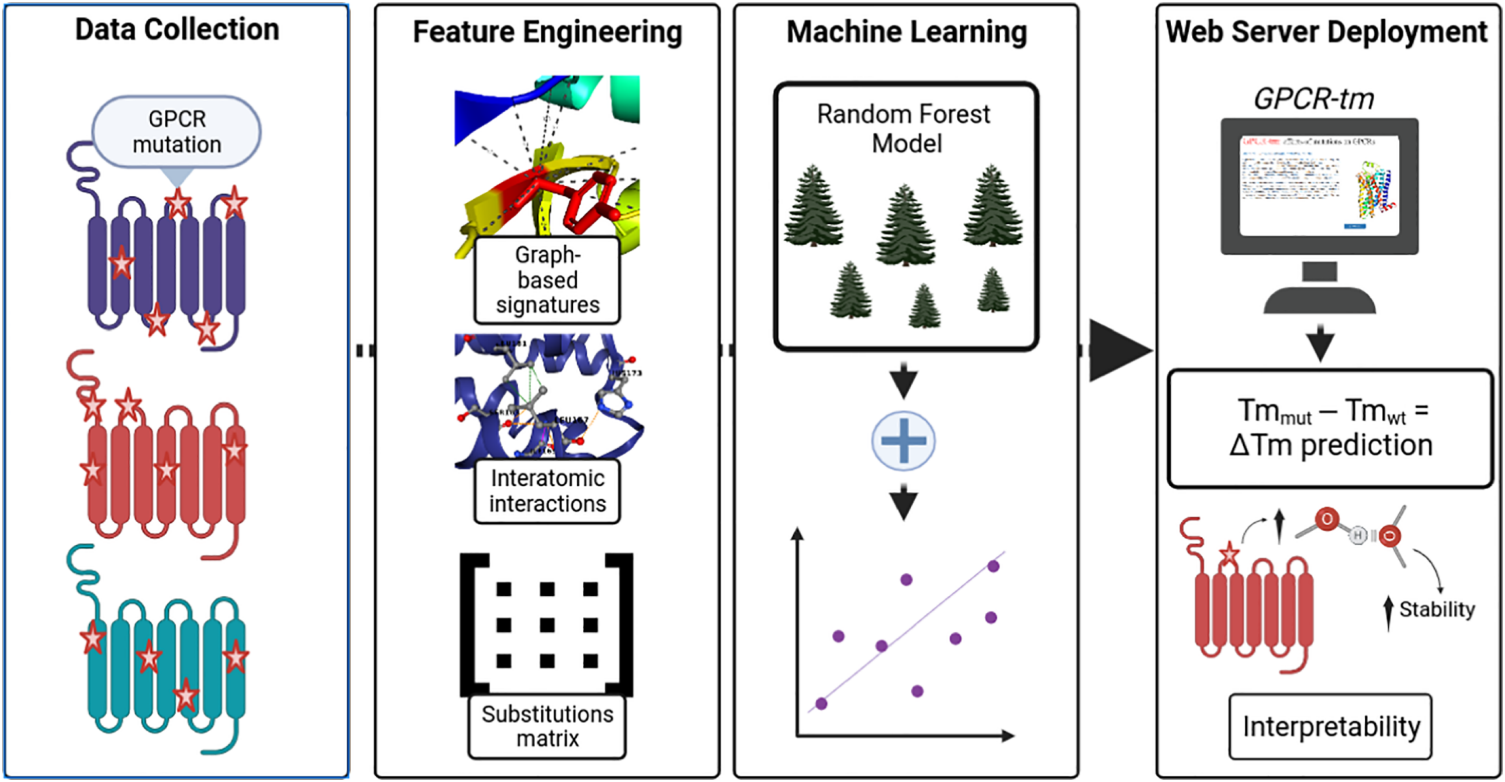
The methodological steps followed in this work: data collection; feature engineering—two main classes of features are generated: (i) graph‐based signatures of the wild‐type residue environment and (ii) auxiliary features, comprehending substitutions matrix, interatomic interactions, and general properties of amino acids; machine learning, where training, validation, optimization and testing of the proposed supervised model are performed; and, web server deployment, which provides easy and robust access to the proposed predictive model (and its interpretation) via a web‐based interface. GPCR, G protein‐coupled receptors.

The general workflow of GPCR‐tm is shown in Figure 3. GPCR‐tm was trained using data sets of experimentally characterized mutations in GPCR proteins, for which structures were available. It is composed of four main steps, including: (i) *data collection*, which refers to collecting experimental data about phenotype‐changing GPCR mutations; (ii) *feature generation*, which encompasses the feature engineering to model different aspects and particularities involved in GPCR data coming from sequences and structures, and (iii) *machine learning*, which highlights the development of the supervised learning models to predict and rank GPCR stabilization based on the computed features and experimental classification of thermal stability upon mutation; (iv) *web server*, which delivers a deployed GPCR‐tm web server for easy and scalable access to the developed ML models from the previous step, providing both GPCR predictions and interpretability via a web platform.

### 4.1 Data collection

We retrieved and gathered experimental ΔTm data from diverse databases, including FireProtDB (Stourac et al., 2021) and Thermomutdb (Xavier et al., 2021). These databases are thorough, manually compiled repositories of protein stability information concerning point mutations. Additionally, we also collected data from MPTherm‐pred (Kulandaisamy et al., 2021), which is a web server that hosts a range of topologically specific models for forecasting the thermal stability of membrane proteins following missense mutations. All datasets were combined, and just one mutation was selected from repeated ones (repeated mutation means same position, same UniProt [Coudert et al., 2022] identification, mutated to the same residue). The mutations across these three databases are summarized in Table 2. The data used to develop GPCR‐tm are freely available to download at https://biosig.lab.uq.edu.au/gpcr_tm/data.

**TABLE 2:** Data collection of G protein‐coupled receptors mutations and their influence on structure stability.

Origin	Number of mutations
FireprotDB (Stourac et al., 2021)	32
MPTherm‐pred (Xavier et al., 2021)	177
Thermomutdb (Kulandaisamy et al., 2021)	16

The generation of features requires wild‐type‐like structures of the receptors represented in the data. We utilized inactive and active states GPCR structure models, which were built using AlphaFold‐Multistate. These GPCR structure models are available in the GPCRdb (Pandy‐Szekeres et al., 2022). All generated models are based on the wild‐type sequence (Pandy‐Szekeres et al., 2022). This step is crucial considering that most of the GPCR structures available on PDB (Berman et al., 2000) contain mutations that are essential for increasing stability and permitting experimental structure elucidation, and it is possible to download models without these mutations on GPCRdb. It is important to note that we selected models without loops. This was done to avoid using regions with low AlphaFold pLDDT scores and reduce structural bias in our proposed ML model (Jumper et al., 2021). We also did a preliminary structure manipulation, removing water molecules, additional chains, and ligands.

### 4.2 Feature engineering

Single‐point mutations have the potential to induce a variety of structural and functional alterations in the protein. Our research focuses on comprehensively capturing and investigating the impact of single‐point mutations on GPCR proteins. To achieve this, we generated both sequence‐ and structure‐based features to provide GPCR‐tm with diverse and complementary variables describing GPCRs (see Table 3). This characterization was subsequently employed to train, validate, and assess the predictive supervised models for predicting stabilization of GPCR proteins.

**TABLE 3:** Sequence‐ and structure‐based features characterizing G protein‐coupled receptors for building the machine learning models.

Type	Name
Sequence‐based	Amino acid substitution scoring matrices (Blosum62, PAM30, and special amino acids)
	Relative solvent accessibility
	Residue depth
	AA index
	Potential function energy calculations
Structure‐based	Arpeggio—molecular interactions
	Structural pattern mining approaches
	Normal mode analysis
	Graph‐based signatures

Abbreviation: AA, amino acid.

#### 4.2.1 Graph‐based structural signatures

One of the main components of our method is a structure‐based feature derived from the concept of graph‐based signatures, which is based on the Cutoff Scanning Matrix algorithm (Pires et al., 2014), which was originally proposed to represent biological systems using network topology by distance patterns.

GPCR‐tm uses a graph‐based representation of the residue environments to extract geometric and physicochemical patterns (the last represented in terms of pharmacophores). The wild‐type residue environment, which here is defined as the set of atoms within a distance *r* from its geometric center, can be modeled as a contact graph, where the atoms are the nodes, and the edges are interactions defined by a cutoff distance. By varying the distance cutoff, different graphs are generated, and cumulative distributions of distances for different interactions are generated, composing a concise and effective representation of the residue environment. To account for the atom changes induced by the mutation, we introduce a *pharmacophore count* vector. Wild‐type and mutant residues are represented as pharmacophore frequency vectors. The frequency of each type of pharmacophore in a residue is then summarized in a vector **p**. The difference (*PChange*) between pharmacophore count for mutant (*pmt*) and wild‐type (*pwt*) residues is calculated and appended to the signature. PChange formula is described in Equation (1):

(1) PChange=pmt–pwt.

The atom pharmacophores are characteristics belonging to eight possible classes: hydrophobic, positive, negative, hydrogen acceptor, hydrogen donor, aromatic, sulfur, and neutral. These signatures have shown to be an effective and efficient method to model protein residue environment, its geometry and physicochemical properties, information that has been used to predict the effects of mutations on protein stability and affinity to its partners (Myung, Pires, & Ascher, 2020; Myung, Rodrigues, et al., 2020; Nguyen et al., 2021; Pires et al., 2014, 2016; Pires & Ascher, 2016, 2017; Rodrigues et al., 2019, 2021a; 2021b, 2024; Rodrigues & Ascher, 2022, 2023; Ryu et al., 2023), pharmacodynamic and pharmacokinetics (Al‐Jarf et al., 2021; de Sa et al., 2022; Iftkhar et al., 2022; Morozov et al., 2023; Pires et al., 2015, 2022; Pires & Ascher, 2020; Rodrigues et al., 2021c, 2022; Velloso et al., 2021), and identify drug resistance (Hawkey et al., 2018; Karmakar et al., 2018, 2019, 2020; Portelli et al., 2020; Portelli, Heaton, & Ascher, 2023; Zhan et al., 2021; Zhou et al., 2021) and disease mutations (Jessen‐Howard et al., 2023; Karmakar et al., 2022; Lai et al., 2021; Portelli et al., 2021; Portelli, Albanaz, et al., 2023).

#### 4.2.2 Auxiliary features

Apart from using the graph‐based signatures to map the structural GPCR protein information, we also employed other complementary features. Inside this set, we have both structure‐based and sequence‐based features.

The sequence‐based feature includes AA substitution scoring matrices (Blosum62, PAM30, and special AAs), relative solvent accessibility (RSA), residue depth (RD), AA index, and potential function energy calculations. More specifically, the AA substitution scoring matrices (Blosum62, PAM30, and special AAs) were used to include information regarding rates at which various AA residues in proteins are substituted by other AA residues over time (Trivedi & Nagarajaram, 2019). RSA of a protein residue, in turn, is a measure of residue solvent exposure (Shrake & Rupley, 1973). Alternatively, RD describes how buried a residue is in the protein structure space (Chakravarty & Varadarajan, 1999). Finally, the AA index (Kawashima et al., 2008) comprehends a set of 20 numerical values representing any of the different physicochemical and biological properties of AAs.

The structure‐based feature includes Arpeggio's molecular interactions, structural pattern mining approaches, and normal mode analysis. Comprehensively, Arpeggio was used to calculate interactions contacts (Jubb et al., 2017), including various types of interactions such as van der Waals, ionic, carbonyl, metal, hydrophobic, and halogen bond contacts, hydrogen bonds, and specific atom–aromatic ring (cation–π, donor–π, halogen–π, and carbon–π) and aromatic ring–aromatic ring (π–π) interactions. We have also included potential function energy calculations used in SDM (Worth et al., 2011), structural pattern mining approaches (e.g., mCSM‐Stability; Pires et al., 2014), and normal mode analysis (by utilizing ENCoM; Frappier & Najmanovich, 2014).

### 4.3 Machine learning

#### 4.3.1 Model building and feature selection

After the generation of features, we aimed to find the best set of features and the best ML algorithm for the building of a reliable regressor and ranking model for GPCR stabilization. We tested four different algorithms (Raschka, 2015): Random Forest, Extremely Randomized Trees, Gradient Boosting, and Extreme Gradient Boosting (XGBOOST). The Scikit‐learn toolkit (Pedregosa et al., 2011) was used for training, (cross‐)validating, and testing the models (Raschka, 2020). All these models used 300 as the number of predictive (decision trees) in their resultant ensemble. Other hyperparameters were set to their respective default Scikit‐learn values.

To avoid overfitting, increase performance, and reduce noise in the data, we attempted to find the best set of features and the best ML. For this task, we used a bottom‐up greedy feature selection algorithm, which is a heuristic algorithm that locally selects the feature with the best feature in terms of a performance metric at each stage. The adopted algorithm employs a forward selection approach. This means it initially starts with zero selected features. Next, it evaluates all features individually and fixes the one with the best score (e.g., Pearson's correlation coefficient), using a 10‐fold cross‐validation procedure on a ML model. Thereafter, all remaining features are tested together with the first one previously selected. Subsequently, this feature selection process continues until the predictive performance stops improving with the inclusion of new features.

The proposed model is a regressor for predicting ΔTm, a continuous value. The blind test selected was 13% of the entire dataset. For testing the predicted values against the actual values, we used the proper metrics for regression models described in the next section. We have also used classification by regression as a means of comparing our tool (predicts ΔTm) with other available tools that predict ΔΔG. Subsequently, we converted the predictions and the actual values for two classes only: mutations that increase stability and mutations that decrease stability. We have also applied classification by regression during our second blind test, when evaluating our model against mutations available at GPCRdb, all characterized as increasing stability (Pandy‐Szekeres et al., 2022). To avoid bias, we just selected mutations that were not included in our training data set. Utilizing a second blind test provides a crucial step for validating the model's generalization and reliability. This was a crucial step in evaluating the prediction ability of GPCR‐tm to capture stabilizing mutations from a broader and generalized mutational landscape. This step was done by converting our prediction values to 0 or 1, meaning destabilizing or stabilizing, respectively. In this approach, zero was considered for negative values, one for positive values, and then the prediction was compared with the actual results. GPCRdb data consisted of 50 stabilizing non‐redundant mutations (245 prior to redundancy removal when compared to the dataset used to train the model) from 16 different GPCRs. The test set contains all types of mutations and proteins from family A.

#### 4.3.2 Predictive performance evaluation

To assess the predictive performance of models, we gauged the effectiveness of our regression predictions by comparing them against both experimental and predicted ΔTm values. To achieve this, we employed Pearson's correlation coefficient and MSE, quantifying the relationships and deviations between our predictions and the actual values. We have also included Kendall's tau metric and the Spearman's rank‐order correlation coefficient to measure the ranking precision of GPCR‐tm. All the evaluation metrics for regression are detailed in Supporting Information S1.

Furthermore, as part of our classification through a regression approach, we evaluated the overall predictive performance of GPCR‐tm using the metrics MCC, accuracy, and weighted F1 score. All the evaluation metrics for classification are also detailed in Supporting Information S1.

Additionally, GPCR‐tm was compared to well‐established tools designed to predict the effects of mutations on protein stability. Because these alternative tools for predicting protein stability are based on ΔΔG values and our tool is based on ΔTm, we compared them using classification by regression. For this purpose, we considered three possible outcomes: mutations that have a destabilizing, neutral, and stabilizing effect. For each one of the assessed predictors, we explored different threshold levels for defining the classification outcomes. We started evaluating neutral as being between a minimum of −0.1 and a maximum of 0.1 (values below −0.1 were considered destabilizing, and values above 0.1 were considered stabilizing). We altered these threshold levels to 0.05 (to the minimum, we subtracted 0.05, and to the maximum, we summed 0.05) until a maximum of −3.0 for the minimum and 3.0 for the maximum.

#### 4.3.3 Model interpretability

In our investigation, we opted to utilize SHAP (Lundberg & Lee, 2017) summary plots to delve into the significance of various features. SHAP assigns an important value to each feature concerning a particular prediction, providing a nuanced understanding of the factors influencing the model's output. These summary plots act as an intuitive and accessible tool, allowing us to comprehend the primary influences shaping the predictions made by GPCR‐tm's model.

Within the SHAP summary plot, the visualization showcases the relationship between feature values and their impact on predictions. It illustrates how both low and high values of features are associated with either stabilization or destabilization effects, contributing to understanding the model's predictions. This visual representation enhances interpretability, making it easier to grasp the intricate dynamics between input features and the model's decision‐making process.

## AUTHOR CONTRIBUTIONS

**David B. Ascher**: Conceptualization; writing – review and editing; supervision; methodology. **João Paulo L. Velloso**: Methodology; data curation; investigation; formal analysis; writing – original draft. **Alex G. C. de Sá**: Methodology; software; validation; writing – review and editing. **Douglas E. V. Pires**: Methodology; writing – review and editing.

## Supporting information


**Data S1.** Supporting Information.

## References

[pro5000-bib-0001] Alhosaini K , Azhar A , Alonazi A , Al‐Zoghaibi F . GPCRs: the most promiscuous druggable receptor of the mankind. Saudi Pharm J. 2021;29:539–551. 34194261 10.1016/j.jsps.2021.04.015 PMC8233523

[pro5000-bib-0002] Al‐Jarf R , de Sa AGC , Pires DEV , Ascher DB . pdCSM‐cancer: using graph‐based signatures to identify small molecules with anticancer properties. J Chem Inf Model. 2021;61:3314–3322. 34213323 10.1021/acs.jcim.1c00168 PMC8317153

[pro5000-bib-0003] Baek M , DiMaio F , Anishchenko I , Dauparas J , Ovchinnikov S , Lee GR , et al. Accurate prediction of protein structures and interactions using a three‐track neural network. Science. 2021;373:871–876. 34282049 10.1126/science.abj8754 PMC7612213

[pro5000-bib-0004] Ballesteros JA , Weinstein H . [19] Integrated methods for the construction of three‐dimensional models and computational probing of structure‐function relations in G protein‐coupled receptors. In: Sealfon SC , editor. Methods in neurosciences. Cambridge: Academic Press; 1995. p. 366–428.

[pro5000-bib-0005] Benner SA , Cohen MA , Gonnet GH . Amino acid substitution during functionally constrained divergent evolution of protein sequences. Protein Eng. 1994;7:1323–1332. 7700864 10.1093/protein/7.11.1323

[pro5000-bib-0006] Berman HM , Westbrook J , Feng Z , Gilliland G , Bhat TN , Weissig H , et al. The Protein Data Bank. Nucleic Acids Res. 2000;28:235–242. 10592235 10.1093/nar/28.1.235 PMC102472

[pro5000-bib-0007] Chakravarty S , Varadarajan R . Residue depth: a novel parameter for the analysis of protein structure and stability. Structure. 1999;7:723–732. 10425675 10.1016/s0969-2126(99)80097-5

[pro5000-bib-0008] Cheng Y . Single‐particle cryo‐EM at crystallographic resolution. Cell. 2015;161:450–457. 25910205 10.1016/j.cell.2015.03.049 PMC4409662

[pro5000-bib-0009] Choi EJ , Mayo SL . Generation and analysis of proline mutants in protein G. Protein Eng Des Sel. 2006;19:285–289. 16549401 10.1093/protein/gzl007

[pro5000-bib-0010] Congreve M , de Graaf C , Swain NA , Tate CG . Impact of GPCR structures on drug discovery. Cell. 2020;181:81–91. 32243800 10.1016/j.cell.2020.03.003

[pro5000-bib-0011] Coudert E , Gehant S , de Castro E , Pozzato M , Baratin D , Neto T , et al. Annotation of biologically relevant ligands in UniProtKB using ChEBI. Bioinformatics. 2022;39:btac793. 10.1093/bioinformatics/btac793 PMC9825770 36484697

[pro5000-bib-0012] de Sa AGC , Long Y , Portelli S , Pires DEV , Ascher DB . toxCSM: comprehensive prediction of small molecule toxicity profiles. Brief Bioinform. 2022;23:bbac337. 35998885 10.1093/bib/bbac337

[pro5000-bib-0013] Errey JC , Fiez‐Vandal C . Production of membrane proteins in industry: the example of GPCRs. Protein Expr Purif. 2020;169:105569. 31945417 10.1016/j.pep.2020.105569

[pro5000-bib-0014] Frank J . Single‐particle imaging of macromolecules by cryo‐electron microscopy. Annu Rev Biophys Biomol Struct. 2002;31:303–319. 11988472 10.1146/annurev.biophys.31.082901.134202

[pro5000-bib-0015] Frappier V , Najmanovich RJ . A coarse‐grained elastic network atom contact model and its use in the simulation of protein dynamics and the prediction of the effect of mutations. PLoS Comput Biol. 2014;10:e1003569. 24762569 10.1371/journal.pcbi.1003569 PMC3998880

[pro5000-bib-0016] Hassani L . Chemical modification of horseradish peroxidase with carboxylic anhydrides: effect of negative charge and hydrophilicity of the modifiers on thermal stability. J Mol Catal B: Enzym. 2012;80:15–19.

[pro5000-bib-0017] Hauser AS , Attwood MM , Rask‐Andersen M , Schioth HB , Gloriam DE . Trends in GPCR drug discovery: new agents, targets and indications. Nat Rev Drug Discov. 2017;16:829–842. 29075003 10.1038/nrd.2017.178 PMC6882681

[pro5000-bib-0018] Hawkey J , Ascher DB , Judd LM , Wick RR , Kostoulias X , Cleland H , et al. Evolution of carbapenem resistance in *Acinetobacter baumannii* during a prolonged infection. Microb Genom. 2018;4:e000165. 29547094 10.1099/mgen.0.000165 PMC5885017

[pro5000-bib-0019] Hirozane Y , Motoyaji T , Maru T , Okada K , Tarui N . Generating thermostabilized agonist‐bound GPR40/FFAR1 using virus‐like particles and a label‐free binding assay. Mol Membr Biol. 2014;31:168–175. 25068810 10.3109/09687688.2014.923588

[pro5000-bib-0020] Iftkhar S , de Sa AGC , Velloso JPL , Aljarf R , Pires DEV , Ascher DB . cardioToxCSM: a web server for predicting cardiotoxicity of small molecules. J Chem Inf Model. 2022;62:4827–4836. 36219164 10.1021/acs.jcim.2c00822

[pro5000-bib-0021] Jessen‐Howard D , Pan Q , Ascher DB . Identifying the molecular drivers of pathogenic aldehyde dehydrogenase missense mutations in cancer and non‐cancer diseases. Int J Mol Sci. 2023;24:10157. 37373306 10.3390/ijms241210157 PMC10299257

[pro5000-bib-0022] Jubb HC , Higueruelo AP , Ochoa‐Montano B , Pitt WR , Ascher DB , Blundell TL . Arpeggio: a web server for calculating and visualising interatomic interactions in protein structures. J Mol Biol. 2017;429:365–371. 27964945 10.1016/j.jmb.2016.12.004 PMC5282402

[pro5000-bib-0023] Jumper J , Evans R , Pritzel A , Green T , Figurnov M , Ronneberger O , et al. Highly accurate protein structure prediction with AlphaFold. Nature. 2021;596:583–589. 34265844 10.1038/s41586-021-03819-2 PMC8371605

[pro5000-bib-0024] Karmakar M , Cicaloni V , Rodrigues CHM , Spiga O , Santucci A , Ascher DB . HGDiscovery: an online tool providing functional and phenotypic information on novel variants of homogentisate 1,2‐ dioxigenase. Curr Res Struct Biol. 2022;4:271–277. 36118553 10.1016/j.crstbi.2022.08.001 PMC9471331

[pro5000-bib-0025] Karmakar M , Globan M , Fyfe JAM , Stinear TP , Johnson PDR , Holmes NE , et al. Analysis of a novel pncA mutation for susceptibility to pyrazinamide therapy. Am J Respir Crit Care Med. 2018;198:541–544. 29694240 10.1164/rccm.201712-2572LE PMC6118032

[pro5000-bib-0026] Karmakar M , Rodrigues CHM , Holt KE , Dunstan SJ , Denholm J , Ascher DB . Empirical ways to identify novel Bedaquiline resistance mutations in AtpE. PLoS One. 2019;14:e0217169. 31141524 10.1371/journal.pone.0217169 PMC6541270

[pro5000-bib-0027] Karmakar M , Rodrigues CHM , Horan K , Denholm JT , Ascher DB . Structure guided prediction of pyrazinamide resistance mutations in pncA. Sci Rep. 2020;10:1875. 32024884 10.1038/s41598-020-58635-x PMC7002382

[pro5000-bib-0028] Kawashima S , Pokarowski P , Pokarowska M , Kolinski A , Katayama T , Kanehisa M . AAindex: amino acid index database, progress report 2008. Nucleic Acids Res. 2008;36:D202–D205. 17998252 10.1093/nar/gkm998 PMC2238890

[pro5000-bib-0029] Kobilka BK , Deupi X . Conformational complexity of G‐protein‐coupled receptors. Trends Pharmacol Sci. 2007;28:397–406. 17629961 10.1016/j.tips.2007.06.003

[pro5000-bib-0030] Kulandaisamy A , Priya SB , Sakthivel R , Frishman D , Gromiha MM . Statistical analysis of disease‐causing and neutral mutations in human membrane proteins. Proteins. 2019;87:452–466. 30714211 10.1002/prot.25667

[pro5000-bib-0031] Kulandaisamy A , Zaucha J , Frishman D , Gromiha MM . MPTherm‐pred: analysis and prediction of thermal stability changes upon mutations in transmembrane proteins. J Mol Biol. 2021;433:166646. 32920050 10.1016/j.jmb.2020.09.005

[pro5000-bib-0032] Lai CY , Tsai IJ , Chiu PC , Ascher DB , Chien YH , Huang YH , et al. A novel deep intronic variant strongly associates with Alkaptonuria. NPJ Genom Med. 2021;6:89. 34686677 10.1038/s41525-021-00252-2 PMC8536767

[pro5000-bib-0033] Laimer J , Hiebl‐Flach J , Lengauer D , Lackner P . MAESTROweb: a web server for structure‐based protein stability prediction. Bioinformatics. 2016;32:1414–1416. 26743508 10.1093/bioinformatics/btv769

[pro5000-bib-0034] Lebon G , Bennett K , Jazayeri A , Tate CG . Thermostabilisation of an agonist‐bound conformation of the human adenosine A_2A_ receptor. J Mol Biol. 2011;409:298–310. 21501622 10.1016/j.jmb.2011.03.075 PMC3145977

[pro5000-bib-0035] Lee S , Ghosh S , Jana S , Robertson N , Tate CG , Vaidehi N . How do branched detergents stabilize GPCRs in micelles? Biochemistry. 2020;59:2125–2134. 32437610 10.1021/acs.biochem.0c00183 PMC7302508

[pro5000-bib-0036] Lundberg S , Lee S‐I . A unified approach to interpreting model predictions. arXiv, 2017; 1705.07874v2.

[pro5000-bib-0037] Luthy R , McLachlan AD , Eisenberg D . Secondary structure‐based profiles: use of structure‐conserving scoring tables in searching protein sequence databases for structural similarities. Proteins. 1991;10:229–239. 1881879 10.1002/prot.340100307

[pro5000-bib-0038] Magnani F , Shibata Y , Serrano‐Vega MJ , Tate CG . Co‐evolving stability and conformational homogeneity of the human adenosine A_2a_ receptor. Proc Natl Acad Sci U S A. 2008;105:10744–10749. 18664584 10.1073/pnas.0804396105 PMC2504806

[pro5000-bib-0039] Matthews BW . Structural and genetic analysis of protein stability. Annu Rev Biochem. 1993;62:139–160. 8352587 10.1146/annurev.bi.62.070193.001035

[pro5000-bib-0040] May LT , Avlani VA , Sexton PM , Christopoulos A . Allosteric modulation of G protein‐coupled receptors. Curr Pharm Des. 2004;10:2003–2013. 15279541 10.2174/1381612043384303

[pro5000-bib-0041] Milligan G , Ward RJ , Marsango S . GPCR homo‐oligomerization. Curr Opin Cell Biol. 2019;57:40–47. 30453145 10.1016/j.ceb.2018.10.007 PMC7083226

[pro5000-bib-0042] Montanucci L , Capriotti E , Birolo G , Benevenuta S , Pancotti C , Lal D , et al. DDGun: an untrained predictor of protein stability changes upon amino acid variants. Nucleic Acids Res. 2022;50:W222–W227. 35524565 10.1093/nar/gkac325 PMC9252764

[pro5000-bib-0043] Morozov V , Rodrigues CHM , Ascher DB . CSM‐toxin: a web‐server for predicting protein toxicity. Pharmaceutics. 2023;15:431. 36839752 10.3390/pharmaceutics15020431 PMC9966851

[pro5000-bib-0044] Munk C , Mutt E , Isberg V , Nikolajsen LF , Bibbe JM , Flock T , et al. An online resource for GPCR structure determination and analysis. Nat Methods. 2019;16:151–162. 30664776 10.1038/s41592-018-0302-x PMC6881186

[pro5000-bib-0045] Myung Y , Pires DEV , Ascher DB . mmCSM‐AB: guiding rational antibody engineering through multiple point mutations. Nucleic Acids Res. 2020;48:W125–W131. 32432715 10.1093/nar/gkaa389 PMC7319589

[pro5000-bib-0046] Myung Y , Rodrigues CHM , Ascher DB , Pires DEV . mCSM‐AB2: guiding rational antibody design using graph‐based signatures. Bioinformatics. 2020;36:1453–1459. 31665262 10.1093/bioinformatics/btz779

[pro5000-bib-0047] Nguyen TB , Myung Y , de Sa AGC , Pires DEV , Ascher DB . mmCSM‐NA: accurately predicting effects of single and multiple mutations on protein‐nucleic acid binding affinity. NAR Genom Bioinform. 2021;3:lqab109. 34805992 10.1093/nargab/lqab109 PMC8600011

[pro5000-bib-0048] Pace CN , Alston RW , Shaw KL . Charge‐charge interactions influence the denatured state ensemble and contribute to protein stability. Protein Sci. 2000;9:1395–1398. 10933506 10.1110/ps.9.7.1395 PMC2144688

[pro5000-bib-0049] Pandurangan AP , Ochoa‐Montano B , Ascher DB , Blundell TL . SDM: a server for predicting effects of mutations on protein stability. Nucleic Acids Res. 2017;45:W229–W235. 28525590 10.1093/nar/gkx439 PMC5793720

[pro5000-bib-0050] Pandy‐Szekeres G , Caroli J , Mamyrbekov A , Kermani AA , Keseru GM , Kooistra AJ , et al. GPCRdb in 2023: state‐specific structure models using AlphaFold2 and new ligand resources. Nucleic Acids Res. 2023;51:D395–D402. 36395823 10.1093/nar/gkac1013 PMC9825476

[pro5000-bib-0051] Park PS , Lodowski DT , Palczewski K . Activation of G protein‐coupled receptors: beyond two‐state models and tertiary conformational changes. Annu Rev Pharmacol Toxicol. 2008;48:107–141. 17848137 10.1146/annurev.pharmtox.48.113006.094630 PMC2639654

[pro5000-bib-0052] Parker JL , Newstead S . Current trends in alpha‐helical membrane protein crystallization: an update. Protein Sci. 2012;21:1358–1365. 22811290 10.1002/pro.2122 PMC3631364

[pro5000-bib-0053] Pedregosa F , Varoquaux G , Gramfort A , Michel V , Thirion B , Grisel O , et al. Scikit‐learn: machine learning in python. J Mach Learn Res. 2011;12:2825–2830.

[pro5000-bib-0054] Pires DE , Ascher DB . mCSM‐AB: a web server for predicting antibody‐antigen affinity changes upon mutation with graph‐based signatures. Nucleic Acids Res. 2016;44:W469–W473. 27216816 10.1093/nar/gkw458 PMC4987957

[pro5000-bib-0055] Pires DEV , Ascher DB . mCSM‐NA: predicting the effects of mutations on protein‐nucleic acids interactions. Nucleic Acids Res. 2017;45:W241–W246. 28383703 10.1093/nar/gkx236 PMC5570212

[pro5000-bib-0056] Pires DEV , Ascher DB . mycoCSM: using graph‐based signatures to identify safe potent hits against mycobacteria. J Chem Inf Model. 2020;60:3450–3456. 32615035 10.1021/acs.jcim.0c00362

[pro5000-bib-0057] Pires DE , Ascher DB , Blundell TL . mCSM: predicting the effects of mutations in proteins using graph‐based signatures. Bioinformatics. 2014;30:335–342. 24281696 10.1093/bioinformatics/btt691 PMC3904523

[pro5000-bib-0058] Pires DE , Blundell TL , Ascher DB . pkCSM: predicting small‐molecule pharmacokinetic and toxicity properties using graph‐based signatures. J Med Chem. 2015;58:4066–4072. 25860834 10.1021/acs.jmedchem.5b00104 PMC4434528

[pro5000-bib-0059] Pires DE , Blundell TL , Ascher DB . mCSM‐lig: quantifying the effects of mutations on protein‐small molecule affinity in genetic disease and emergence of drug resistance. Sci Rep. 2016;6:29575. 27384129 10.1038/srep29575 PMC4935856

[pro5000-bib-0060] Pires DEV , Rodrigues CHM , Ascher DB . mCSM‐membrane: predicting the effects of mutations on transmembrane proteins. Nucleic Acids Res. 2020;48:W147–W153. 32469063 10.1093/nar/gkaa416 PMC7319563

[pro5000-bib-0061] Pires DEV , Stubbs KA , Mylne JS , Ascher DB . cropCSM: designing safe and potent herbicides with graph‐based signatures. Brief Bioinform. 2022;23:bbac042. 35211724 10.1093/bib/bbac042 PMC9155605

[pro5000-bib-0062] Portelli S , Albanaz A , Pires DEV , Ascher DB . Identifying the molecular drivers of ALS‐implicated missense mutations. J Med Genet. 2023;60:484–490. 36180205 10.1136/jmg-2022-108798

[pro5000-bib-0063] Portelli S , Barr L , de Sa AGC , Pires DEV , Ascher DB . Distinguishing between PTEN clinical phenotypes through mutation analysis. Comput Struct Biotechnol J. 2021;19:3097–3109. 34141133 10.1016/j.csbj.2021.05.028 PMC8180946

[pro5000-bib-0064] Portelli S , Heaton R , Ascher DB . Identifying innate resistance hotspots for SARS‐CoV‐2 antivirals using in silico protein techniques. Genes. 2023;14:1699. 37761839 10.3390/genes14091699 PMC10531314

[pro5000-bib-0065] Portelli S , Myung Y , Furnham N , Vedithi SC , Pires DEV , Ascher DB . Prediction of rifampicin resistance beyond the RRDR using structure‐based machine learning approaches. Sci Rep. 2020;10:18120. 33093532 10.1038/s41598-020-74648-y PMC7581776

[pro5000-bib-0066] Raschka S . Python machine learning. Birmingham: Packt Publishing; 2015.

[pro5000-bib-0067] Raschka S (2020) Model evaluation, model selection, and algorithm selection in machine learning.

[pro5000-bib-0068] Rodrigues CHM , Ascher DB . CSM‐potential: mapping protein interactions and biological ligands in 3D space using geometric deep learning. Nucleic Acids Res. 2022;50:W204–W209. 35609999 10.1093/nar/gkac381 PMC9252741

[pro5000-bib-0069] Rodrigues CHM , Ascher DB . CSM‐Potential2: a comprehensive deep learning platform for the analysis of protein interacting interfaces. Proteins. 2023. 10.1002/prot.26615 PMC11623435 37870486

[pro5000-bib-0070] Rodrigues CHM , Garg A , Keizer D , Pires DEV , Ascher DB . CSM‐peptides: a computational approach to rapid identification of therapeutic peptides. Protein Sci. 2022;31:e4442. 36173168 10.1002/pro.4442 PMC9518225

[pro5000-bib-0071] Rodrigues CHM , Myung Y , Pires DEV , Ascher DB . mCSM‐PPI2: predicting the effects of mutations on protein‐protein interactions. Nucleic Acids Res. 2019;47:W338–W344. 31114883 10.1093/nar/gkz383 PMC6602427

[pro5000-bib-0072] Rodrigues CH , Pires DE , Ascher DB . DynaMut: predicting the impact of mutations on protein conformation, flexibility and stability. Nucleic Acids Res. 2018;46:W350–W355. 29718330 10.1093/nar/gky300 PMC6031064

[pro5000-bib-0073] Rodrigues CHM , Pires DEV , Ascher DB . DynaMut2: assessing changes in stability and flexibility upon single and multiple point missense mutations. Protein Sci. 2021a;30:60–69. 32881105 10.1002/pro.3942 PMC7737773

[pro5000-bib-0074] Rodrigues CHM , Pires DEV , Ascher DB . mmCSM‐PPI: predicting the effects of multiple point mutations on protein‐protein interactions. Nucleic Acids Res. 2021b;49:W417–W424. 33893812 10.1093/nar/gkab273 PMC8262703

[pro5000-bib-0075] Rodrigues CHM , Pires DEV , Ascher DB . pdCSM‐PPI: using graph‐based signatures to identify protein‐protein interaction inhibitors. J Chem Inf Model. 2021c;61:5438–5445. 34719929 10.1021/acs.jcim.1c01135

[pro5000-bib-0076] Rodrigues CHM , Portelli S , Ascher DB . Exploring the effects of missense mutations on protein thermodynamics through structure‐based approaches: findings from the CAGI6 challenges. Hum Genet. 2024. 10.1007/s00439-023-02623-4 PMC11976750 38227011

[pro5000-bib-0077] Ryu J , Barkal S , Yu T , Jankowiak M , Zhou Y , Francoeur M , et al. Joint genotypic and phenotypic outcome modeling improves base editing variant effect quantification. medRxiv. 2023. 10.1038/s41588-024-01726-6 PMC11669423 38658794

[pro5000-bib-0078] Shibata Y , Gvozdenovic‐Jeremic J , Love J , Kloss B , White JF , Grisshammer R , et al. Optimising the combination of thermostabilising mutations in the neurotensin receptor for structure determination. Biochim Biophys Acta. 2013;1828:1293–1301. 23337476 10.1016/j.bbamem.2013.01.008 PMC3582860

[pro5000-bib-0079] Shibata Y , White JF , Serrano‐Vega MJ , Magnani F , Aloia AL , Grisshammer R , et al. Thermostabilization of the neurotensin receptor NTS1. J Mol Biol. 2009;390:262–277. 19422831 10.1016/j.jmb.2009.04.068 PMC2696590

[pro5000-bib-0080] Shrake A , Rupley JA . Environment and exposure to solvent of protein atoms. Lysozyme and insulin. J Mol Biol. 1973;79:351–371. 4760134 10.1016/0022-2836(73)90011-9

[pro5000-bib-0081] Stourac J , Dubrava J , Musil M , Horackova J , Damborsky J , Mazurenko S , et al. FireProtDB: database of manually curated protein stability data. Nucleic Acids Res. 2021;49:D319–D324. 33166383 10.1093/nar/gkaa981 PMC7778887

[pro5000-bib-0082] Trivedi R , Nagarajaram HA . Amino acid substitution scoring matrices specific to intrinsically disordered regions in proteins. Sci Rep. 2019;9:16380. 31704957 10.1038/s41598-019-52532-8 PMC6841959

[pro5000-bib-0083] Vaidehi N , Grisshammer R , Tate CG . How can mutations thermostabilize G‐protein‐coupled receptors? Trends Pharmacol Sci. 2016;37:37–46. 26547284 10.1016/j.tips.2015.09.005 PMC4698185

[pro5000-bib-0084] Velloso JPL , Ascher DB , Pires DEV . pdCSM‐GPCR: predicting potent GPCR ligands with graph‐based signatures. Bioinform Adv. 2021;1:vbab031. 34901870 10.1093/bioadv/vbab031 PMC8651072

[pro5000-bib-0085] Wootten D , Christopoulos A , Marti‐Solano M , Babu MM , Sexton PM . Mechanisms of signalling and biased agonism in G protein‐coupled receptors. Nat Rev Mol Cell Biol. 2018;19:638–653. 30104700 10.1038/s41580-018-0049-3

[pro5000-bib-0086] Worth CL , Preissner R , Blundell TL . SDM – a server for predicting effects of mutations on protein stability and malfunction. Nucleic Acids Res. 2011;39:W215–W222. 21593128 10.1093/nar/gkr363 PMC3125769

[pro5000-bib-0087] Xavier JS , Nguyen TB , Karmarkar M , Portelli S , Rezende PM , Velloso JPL , et al. ThermoMutDB: a thermodynamic database for missense mutations. Nucleic Acids Res. 2021;49:D475–D479. 33095862 10.1093/nar/gkaa925 PMC7778973

[pro5000-bib-0088] Zhan J , Harrison AR , Portelli S , Nguyen TB , Kojima I , Zheng S , et al. Definition of the immune evasion‐replication interface of rabies virus P protein. PLoS Pathog. 2021;17:e1009729. 34237115 10.1371/journal.ppat.1009729 PMC8291714

[pro5000-bib-0089] Zhou Y , Pan Q , Pires DEV , Rodrigues CHM , Ascher DB . DDMut: predicting effects of mutations on protein stability using deep learning. Nucleic Acids Res. 2023;51:W122–W128. 37283042 10.1093/nar/gkad472 PMC10320186

[pro5000-bib-0090] Zhou Y , Portelli S , Pat M , Rodrigues CHM , Nguyen TB , Pires DEV , et al. Structure‐guided machine learning prediction of drug resistance mutations in Abelson 1 kinase. Comput Struct Biotechnol J. 2021;19:5381–5391. 34667533 10.1016/j.csbj.2021.09.016 PMC8495037

