# Supplementary material for: Engineering G protein‐coupled receptors for stabilization
Source: Protein Sci. 2024 May 15;33(6):e5000. doi: 10.1002/pro.5000 (PMC11094779; doi:10.1002/pro.5000)
Supplement: Supplementary file 1 — Data S1. Supporting Information. [file PRO-33-e5000-s001.docx]

**SUPPLEMENTARY MATERIALS**

**Engineering G protein-coupled receptors for stabilisation**

João Paulo L. Velloso^1,2,3^, Alex G. C. de Sá^1,2,3^, Douglas E.V. Pires^4^, David B. Ascher^1,2,3^

^1^ School of Chemistry and Molecular Biosciences, The University of Queensland, Brisbane, Queensland, 4072, Australia

^2^ Computational Biology and Clinical Informatics, Baker Heart and Diabetes Institute, Melbourne, Victoria, 3004, Australia

^3^ Baker Department of Cardiometabolic Health, The University of Melbourne, Parkville, Victoria, 3010, Australia

^4^ School of Computing and Information Systems, The University of Melbourne, Parkville, Victoria, 3052, Australia

*To whom correspondence should be addressed D.B.A. Tel: +61 7 336 53991; Email: [d.ascher@uq.edu.au](mailto:david.ascher@unimelb.edu.au).

**SUPPLEMENTARY METHODS**

**Evaluation metrics for regression models**

**Pearson’s Correlation Coefficient (r).** Pearson’s correlation coefficient serves as a common metric for assessing regression models. It measures the extent of linear correlation between the actual and predicted target variables. The values of r range from -1.0 to +1.0, where -1.0 and +1.0 indicate perfect linear relationships between two variables. Pearson’s correlation coefficient is defined in the following equation:


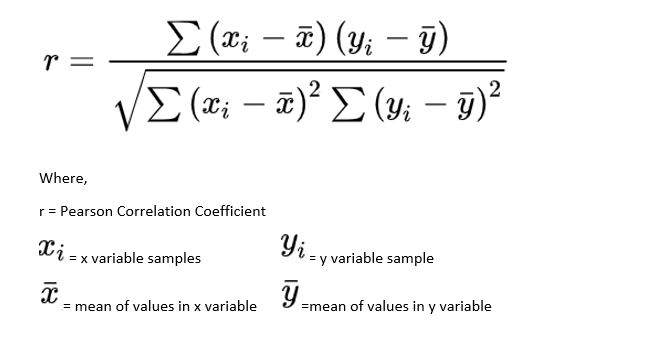


**Mean squared error (MSE).** MSE estimates the errors of the model’s predictions, by measuring the average of the squares of the errors. In other words, the average squared difference between the predicted values and the observed value. MSE is presented in the following equation**:**


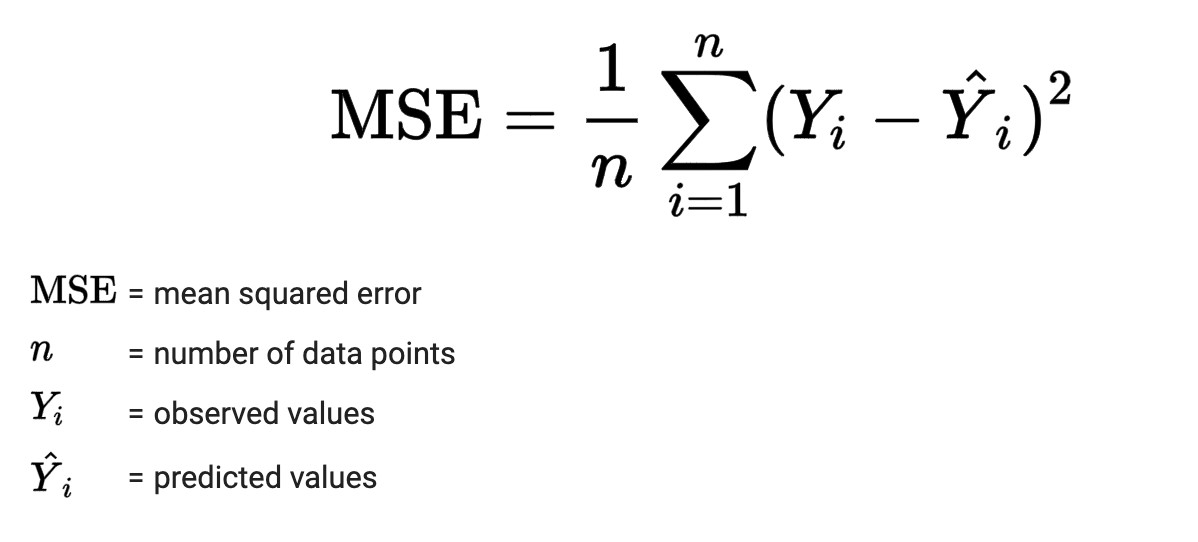


**Kendall’s tau metric.** Kendall’s tau is a measure of the correspondence between two rankings. Values close to 1 indicate strong agreement, values close to -1 indicate strong disagreement. The definition of Kendall’s tau that is used is:

tau = (P - Q) / √ ((P + Q + T) * (P + Q + U)),

where P is the number of concordant pairs, Q is the number of discordant pairs, T is the number of ties only in x, and U is the number of ties only in y. If a tie occurs for the same pair in both x and y, it is not added to either T or U.

**The Spearman’s rank-order correlation coefficient.** The Spearman’s rank-order correlation coefficient is a nonparametric measure of the monotonicity of the relationship between two datasets. Like other correlation coefficients, this one varies between -1 and +1. A value of +1 means a perfect association of rank. A value of 0 means that there is no association between ranks. A value of -1 means a perfect negative association of rank.


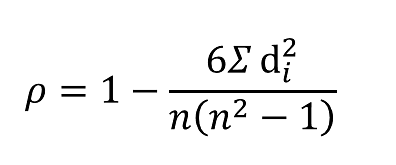


𝝆 = Spearman’s rank correlation coefficient

d_i_ = Difference between the two ranks of each observation

n = Number of observations

**Evaluation metrics for classification models**

**Matthews correlation coefficient (MCC).** MCC is a crucial metric in assessing classification models, especially when dealing with imbalanced datasets. MCC considers both false and true positive and negative predictions to offer a comprehensive evaluation of imbalanced learning. Functioning as a correlation coefficient, MCC ranges from -1 to +1. A score of +1 indicates a perfect positive correlation, while -1 signifies an inverse correlation between the predictions and the true class labels. Meanwhile, a score of 0 suggests a performance equivalent to a random classifier. MCC is determined in the following equation:


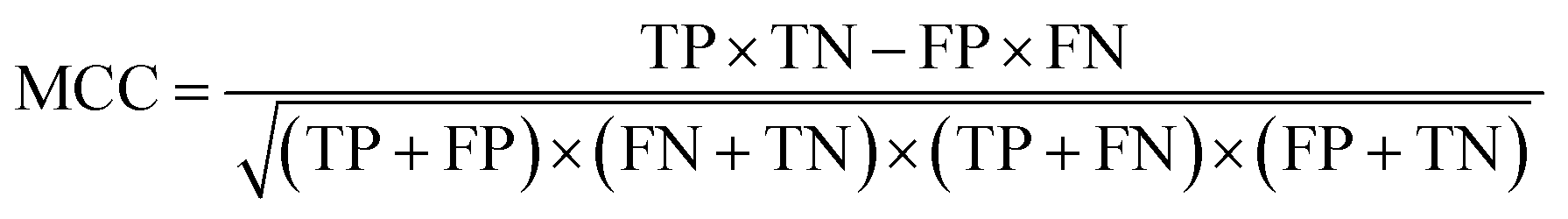


Here, TP stands for True Positives, TN for True Negatives, FP for False Positives, and FN for False Negatives.

**Accuracy.** Accuracy is a measure that gauges the overall correctness of a classification model by considering the ratio of correctly predicted instances to the total instances. The accuracy formula is expressed as:


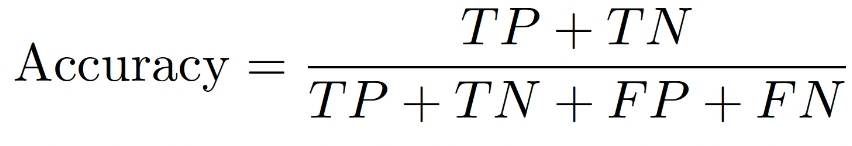


**F1 Score**. The F1-score is a metric that combines precision and recall, giving consideration to both false positives and false negatives, with a focus on imbalanced datasets. It calculates the harmonic mean of precision and recall, assigning different weights to each class based on their prevalence in the dataset. The formula for the F1 Score is given by:


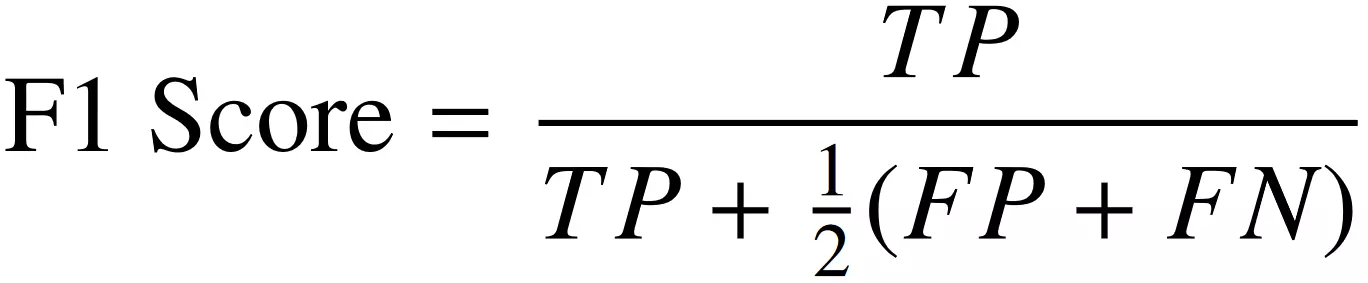


**Weighted F1 Score**. Here, we employed a weighted version of the F1 score, presented in the following equation. In this equation, $N$ represents the number of classes, $F1 Score$_i_ is the F1-score for class $i$ and ${Wi}$is the weight assigned to the class $i$, usually determined by the class distribution in the dataset. The Weighted F1 Score provides a more nuanced evaluation of a model's performance, particularly in scenarios where class imbalances exist.


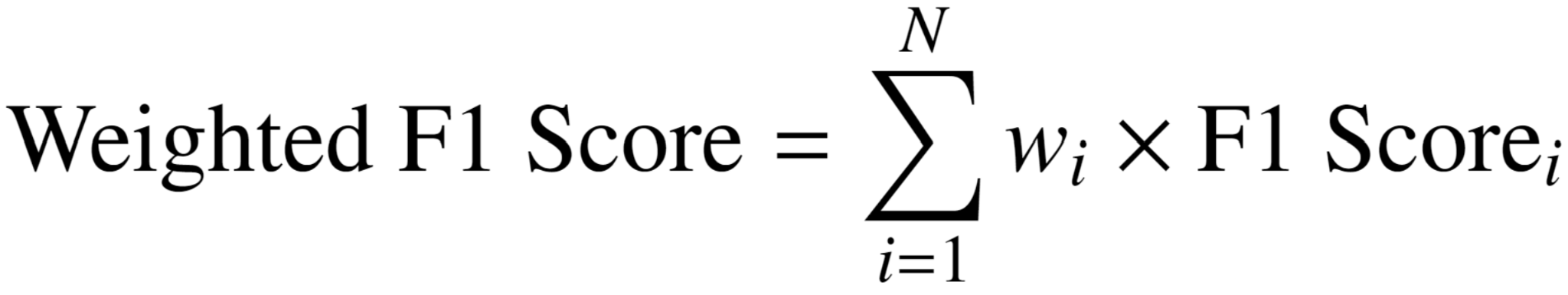


**SUPPLEMENTARY TABLES**

**Table S1. Selected features by the feature selection method and employed by GPCRtm’s final predictive model.**

|  | **Feature ID** | **Description** | **Reference** |
| --- | --- | --- | --- |
| 1 | LUTR910108 | Structure-based comparison table for alpha-helix class. | Luthy et al., 1991 |
| 2 | Hydro:Hydro-4.00 | Graph-based signature: Presence of pharmacophoric pairs Hydrophobic: Hydrophobic in a distance cut-off of 4 angstroms. | Pires et al., 2014 |
| 3 | Don:Pos-5.50 | Graph-based signature: Presence of pharmacophoric pairs Hydrogen bond donor: Positive in a distance cut-off of 5.50 angstroms. | Pires et al., 2014 |
| 4 | Acc:Hydro-2.50 | Graph-based signature: Presence of pharmacophoric pairs Hydrogen bond acceptor: Hydrophobic in a distance cut-off of 5.50 angstroms. | Pires et al., 2014 |
| 5 | Hydro:Pos-5.00 | Graph-based signature: Presence of pharmacophoric pairs Hydrophobic: Positive group in a distance cut-off of 5 angstroms. | Pires et al., 2014 |
| 6 | BENS940104 | Genetic code matrix. | Benner et al., 1994 |
| 7 | mem | Mutation occurring in the membrane. |  |
| 8 | Hydro:Sul-3.50 | Graph-based signature: Presence of pharmacophoric pairs Hydrophobic: Sulphur group in a distance cut-off of 3.50 angstroms. | Pires et al., 2014 |
| 9 | Don:Sul-5.50 | Graph-based signature: Presence of pharmacophoric pairs Hydrogen bond donor: Sulphur group in a distance cut-off of 5.50 angstroms | Pires et al., 2014 |
| 10 | non_cytosol | Mutation occurring outside the cytosol. |  |
| 11 | Hydro:Sul-6.00 | Graph-based signature: Presence of pharmacophoric pairs Hydrophobic: Sulphur group in a distance cut-off of 6 angstroms. | Pires et al., 2014 |
| 12 | Hydro:Pos-3.50 | Graph-based signature: Presence of pharmacophoric pairs Hydrophobic: positive group in a distance cut-off of 3.50 angstroms. | Pires et al., 2014 |
| 13 | FromPRO | Mutation from proline to another amino acid |  |
| 14 | Aro:Neg-4.50 | Graph-based signature: Presence of pharmacophoric pairs Aromatic: negative group in a distance cut-off of 4.50 angstroms. | Pires et al., 2014 |

**SUPPLEMENTARY FIGURES**


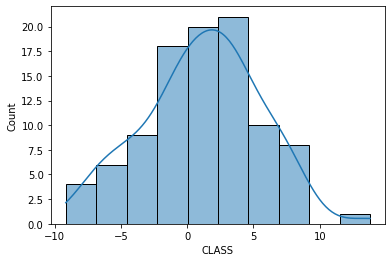


**Figure S1:**  Histogram depicting the distribution of change in melting temperatures (ΔTm), x-axis, caused by mutations in GPCRs.


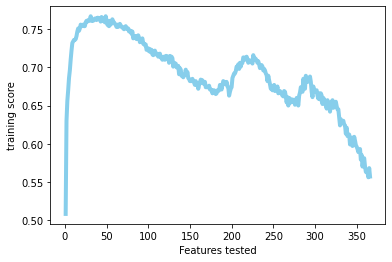


**Figure S2.**  Learning plot curve of the forward feature selection. The y-axis indicates the predictive performance (in terms of Pearson’s correlation coefficient), and the x-axis shows the number of features tested.

**
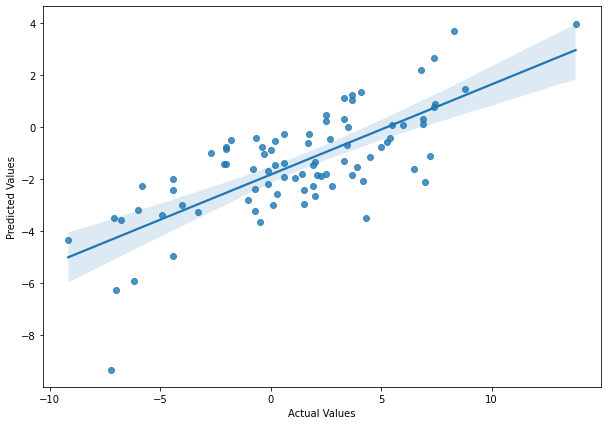
**

**Figure S3.** The regression analysis, which is based on using a 10-fold cross-validation procedure. We analysed the performance of our model through 10-fold cross-validation. The plot shows the high correlation between experimental and predicted values.


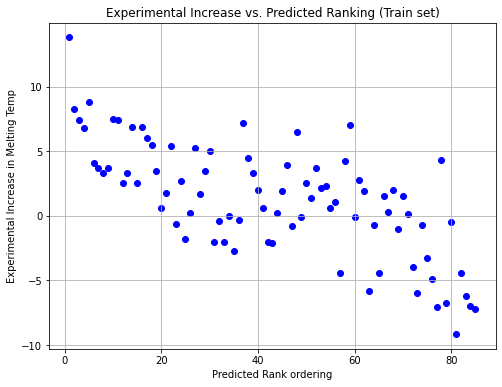


**Figure S4. Scatter plot, experimental ΔTm versus predicted ranking on 10-fold cross-validation:** This scatter plot presents the relationship between the experimentally measured thermal stability (ΔTm) resulting from mutations and their respective rank ordering as predicted by our computational model. Each data point represents a specific mutation within a GPCR sequence in the 10-fold cross-validation, with the x-axis indicating the predicted rank order of mutations by the model and the y-axis representing the experimentally determined ΔTM in degrees Celsius (°C).


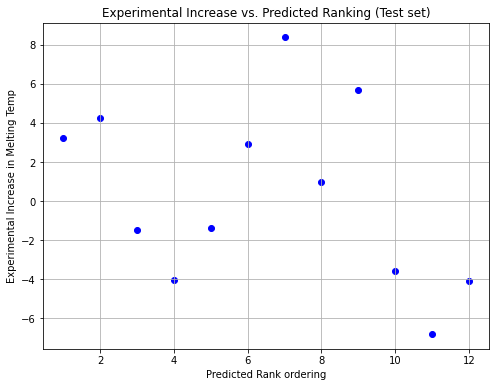


**Figure S5. Scatter plot, experimental ΔTm versus predicted ranking on the blind test set:** This scatter plot presents the relationship between the experimentally measured thermal stability (ΔTm) resulting from mutations and their respective rank ordering as predicted by our computational model. Each data point represents a specific mutation within a GPCR sequence in the blind test set, with the x-axis indicating the predicted rank order of mutations by the model and the y-axis representing the experimentally determined ΔTM in degrees Celsius (°C).

**
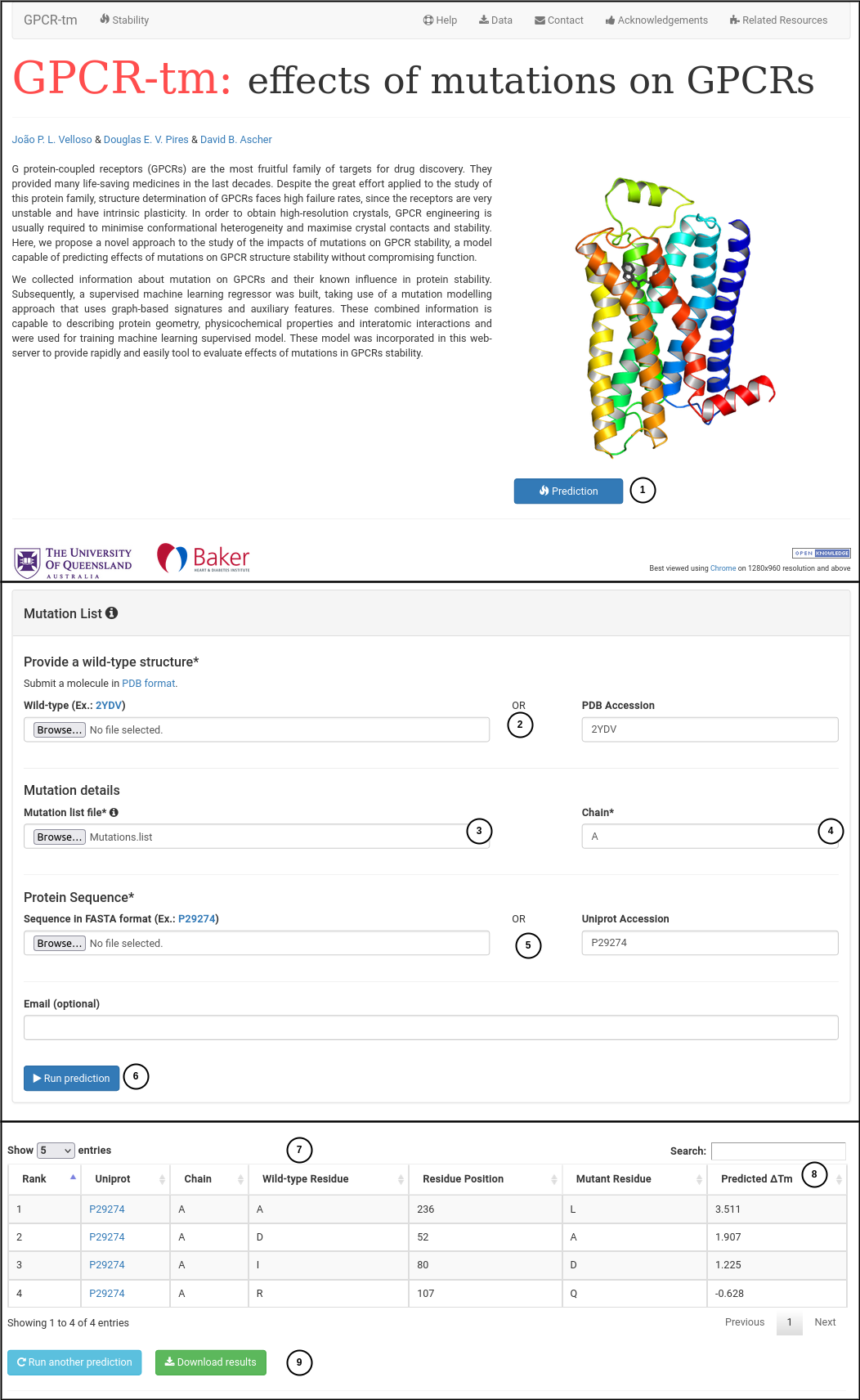
**

**Figure S6.** GPCR-tm web-based interface. For running a prediction, click on *Stability* on the top of the first page (1). Provide the structure of the wild-type protein, which must comply with the PDB format, by either providing a PDB accession code or by uploading your own structure (2). A file with a list of mutations to be analysed should be provided (3), with one mutation per line, following the aforementioned mutation format. The chain must also be provided and consistent with the PDB file (4). Users must provide the UniProt accession code or fasta file of their protein (5). When all the information is completed and the user is ready to submit their query, click *Run prediction* for analysis (6). Your results for a list of mutations will be displayed in a table format with the following information: Mutation information (7), the predicted effect (ΔH) (8). It is also possible to download the predictions as a tab-separated file is also available (9).
